# Exploring drug-target interaction networks of illicit drugs

**DOI:** 10.1186/1471-2164-14-S4-S1

**Published:** 2013-10-01

**Authors:** Ravi V Atreya, Jingchun Sun, Zhongming Zhao

**Affiliations:** 1 Department of Biomedical Informatics, Vanderbilt University School of Medicine, Nashville, TN 37203, USA; 2 Department of Psychiatry, Vanderbilt University School of Medicine, Nashville, TN 37212, USA; 3 Department of Cancer Biology, Vanderbilt University Medical Center, Nashville, TN 37232, USA

## Abstract

**Background:**

Drug addiction is a complex and chronic mental disease, which places a large burden on the American healthcare system due to its negative effects on patients and their families. Recently, network pharmacology is emerging as a promising approach to drug discovery by integrating network biology and polypharmacology, allowing for a deeper understanding of molecular mechanisms of drug actions at the systems level. This study seeks to apply this approach for investigation of illicit drugs and their targets in order to elucidate their interaction patterns and potential secondary drugs that can aid future research and clinical care.

**Results:**

In this study, we extracted 188 illicit substances and their related information from the DrugBank database. The data process revealed 86 illicit drugs targeting a total of 73 unique human genes, which forms an illicit drug-target network. Compared to the full drug-target network from DrugBank, illicit drugs and their target genes tend to cluster together and form four subnetworks, corresponding to four major medication categories: depressants, stimulants, analgesics, and steroids. External analysis of Anatomical Therapeutic Chemical (ATC) second sublevel classifications confirmed that the illicit drugs have neurological functions or act via mechanisms of stimulants, opioids, and steroids. To further explore other drugs potentially having associations with illicit drugs, we constructed an illicit-extended drug-target network by adding the drugs that have the same target(s) as illicit drugs to the illicit drug-target network. After analyzing the degree and betweenness of the network, we identified hubs and bridge nodes, which might play important roles in the development and treatment of drug addiction. Among them, 49 non-illicit drugs might have potential to be used to treat addiction or have addictive effects, including some results that are supported by previous studies.

**Conclusions:**

This study presents the first systematic review of the network characteristics of illicit drugs, their targets, and other drugs that share the targets of these illicit drugs. The results, though preliminary, provide some novel insights into the molecular mechanisms of drug addiction. The observation of illicit-related drugs, with partial verification from previous studies, demonstrated that the network-assisted approach is promising for the identification of drug repositioning.

## Background

Drug addiction is a complex and chronic mental disease characterized by compulsive drug seeking and use despite its harmful consequences. It places a large burden on the American healthcare system due to the detrimental effects of this disease and associated drug use on patients and their families. Abuse and addiction to illicit drugs leads to increased rates of illness [1] and emergency room treatment [2,3]. Additionally, substance abuse rates are higher in patients with psychiatric illness (e.g., schizophrenia), which leads to an increase in the risk of violent and aggressive behavior [4].

Over the last few decades, investigators studying addiction behavior have identified numerous genetic and environmental factors that contribute to the development of addiction. Twin and familial studies have suggested that the genetic factors account for 30-60% of the overall risk for the development of drug addiction [5]. This genetic involvement has been shown to be polygenic, which involves multiple genes that might cooperate through numerous pathways [6]. In addition to addiction, many psychiatric ailments related to illicit drug use are also polygenic and have complex genetic causes [7]. Much of the research on addiction has been general or focused on only one individual drug but not on a category of drugs. Familial analyses [8,9] and literature reviews [10,11] have been the primary drivers of research in this domain. As pharmaceuticals are and will continue to be a driver of care for addiction and other related psychiatric illness, further work is needed to focus on addiction from the perspective of the illicit substances and medications used to treat the addictive effects [12]. Due to the genetic complexity of addiction and the numerous types of addictive substances, we hypothesize that a large-scale analysis of multiple illicit drugs and their target genes can provide a broader understanding of substance abuse.

Recently, network pharmacology approaches have been utilized to transition drug discovery techniques from developing ligands for single targets to more clinically efficacious drugs that target multiple proteins [13-17]. Application of these concepts in the investigation of the drugs range from target identification and side effect prediction [18] to analyses of protein interactions and molecular transport [5]. These studies help researchers and clinicians understand molecular mechanisms, treatment indications, and side effects of antipsychotic medications and, thus, network pharmacology has become a new paradigm in drug discovery [19,20]. In the drug-target interaction networks, nodes can represent drugs, diseases, targets, and proteins while the edges represent physical interactions and regulations between nodes. Network-based approaches might be vital in understanding the genetics and substances involved in addiction. However, this approach relies upon a complete set of drug and drug target data. Recently, DrugBank, a publicly available database, has provided a comprehensive set of literature-extracted bioinformatics and cheminformatics data [21-23], which makes it possible to study all addiction-related drugs and their targets at the systems level.

In this study, we extended our previous, preliminary work [24] and applied network pharmacology approaches for analysis of illicit drugs and their targets to further understand the relationships between the various addictive substances and their targets. To identify drugs that have the potential to cause addictive effects or be used to treat addiction, we further explored the drugs that have common targets with illicit drugs in the context of drug-target networks. This work is novel because it is first to apply a network-based approach to revealing the relationship between illicit drugs and their targets in order to better understand the processes of addiction. Moreover, this network analysis could identify some important targets and drugs, which might enhance the development of treatment strategies for addiction.

## Materials and methods

### Drugs and their corresponding targets

To obtain information on drugs and their targets, we accessed the XML drug data from DrugBank (version 3.0) [25], which includes 6712 drugs and 150 data fields for each drug. Half of this information from DrugBank is devoted to drug/chemical data and the other half is devoted to drug target or protein data. It is one of the most comprehensive repositories of biochemical and genetic information for drugs from peer-reviewed literature. The detailed information on drugs and their targets allows for systems analyses of multiple drugs and their targets simultaneously at the molecular level.

In this study, we focused specifically on illicit drugs and selected those drugs with "illicit" annotation in the "Group" field. In DrugBank, the "Group" field represents the legal status of a drug while "Category" represents the specific class of the medication based on therapeutic characteristics. For each drug, we extracted information from the following fields: "Name," "Accession Number," "Groups," "Categories," "ATC Codes," and "Targets." In the "Targets" field, the data contains drug targets to which a drug can bind, including proteins, macromolecules, nucleic acids, or small molecules. We only extracted human proteins with UniProtKB identifiers and then mapped them to Entrez gene symbols and IDs by using the UniProt ID Mapping service [26]. To investigate if some non-illicit drugs might have addictive effects or could be used to treat addiction, we further collected a set of non-illicit drugs that share at least one common target with at least one illicit drug. The set of drugs were grouped as illicit-related drugs. For the purpose of comparison, we also collected a set of all approved medications with targets and their targets in DrugBank. After excluding illicit drugs and illicit-related drugs, we grouped the remaining drugs as "other drugs." Therefore, we utilized four sets of drugs: illicit drugs, illicit-related drugs, other drugs, and all drugs.

To further examine the classification characteristics of illicit drugs and illicit-related drugs compared to other drugs, we utilized the Anatomical Therapeutic Chemical (ATC) classification system [27]. The ATC system is controlled by the WHO Collaborating Centre for Drug Statistics Methodology. The system groups active drugs into five different levels according to the organ or system on which they act as well as their therapeutic and chemical characteristics. The first level of the ATC classification has fourteen anatomical main groups, of which each is represented by one letter. For example, N represents "nervous system." The second sublevel of the ATC coding system contains systems-specific therapeutic subgroups represented by a two-digit number. For example, N05 represents "psycholeptics," a therapeutic subgroup of the anatomical group N "nervous system." In this study, we utilized the second sublevels of drug classification.

To compare the ATC classification characteristics of the two sets of drugs (illicit drugs or illicit-related drugs) to other drugs, we performed Fisher's exact test for each drug ATC second sublevel category. It should be noted that other drugs are defined as all drugs having at least one target from DrugBank and excluding illicit drugs and illicit-related drugs. For each drug category, we constructed a 2 × 2 contingency table, which includes four counts: n,N-n,r,R-r where n  is the number of illicit drugs or illicit-related drugs in the category, N  is the number of other drugs belonging to the category, r  is the number of illicit drugs or illicit-related drugs not belonging to the category, and R  is the number of other drugs not belonging to this category. Therefore, for each ATC second sublevel category, we calculated one p-value and then chose the ATC second sublevel with a p-value that was less than 0.05 as the significant category compared to other drugs with targets.

### Functional analysis

To characterize the functionality of drugs' target genes, we performed Gene Ontology (GO) Slim Classification and Kyoto Encyclopedia of Genes and Genomes (KEGG) enrichment analyses of drugs' target genes by using the Web-Based Gene Set Analysis Toolkit (WebGestalt) [28]. For GO Slim Classification analysis, WebGestalt maps a set of genes to a cut-down version of the GO terms and counts numbers of the interest genes existing in the corresponding gene set of a given GO Slim term. We first input the illicit drugs' target genes and non-illicit drugs' target genes into WebGestalt, respectively, to obtain the numbers of genes observed in each category and then conducted Fisher's exact test. Here, we performed the enrichment analysis using terms from two GO domains: Biological Process (BP) and Molecular Function (MF). For KEGG pathway analysis, WebGestalt overlays a set of genes to human KEGG pathways via statistical test to identify the pathways enriched with the drugs' target genes. WebGestalt applies a hypergeometric test followed by correction of Benjamini-Hochberg method to reduce type I error, thus, it calculates adjusted p-values after multiple test correction [29]. In this study, we applied an adjusted p-value cutoff 0.05 to define KEGG pathways that are significantly enriched with illicit drugs' target genes or non-illicit drugs' target genes, and then performed Fisher's exact test for statistical significance.

To compare these GO Slim terms and enriched KEGG pathways for illicit drugs' target genes with those of all non-illicit drugs' target genes, we adopted a design similar to a specific statistical test above, i.e., Fisher's exact test. For each GO Slim term or KEGG pathway, we constructed a 2 × 2 contingency table, which includes four counts: n,N-n,r,R-r where n  is the number of drug target genes in the GO Slim term or KEGG pathway, N  is the number of other drug target genes in the DrugBank belonging to the same GO Slim term or KEGG pathway, r  is the number of illicit drug target genes not belonging to the GO Slim term or KEGG pathway, and R  is the number of other drug targets that not belonging to the GO Slim term or KEGG pathway. Therefore, for each GO Slim term or KEGG pathway, we calculated one p-value and chose the GO Slim term or KEGG pathway with a p-value that was less than 0.05 as the significant GO Slim term or KEGG pathway.

### Network analysis

In our drug-target networks, the nodes are drugs and target genes while the edges represent the interactions between drugs and their targets. Based on the sets of drugs and their target genes collected above, we built three drug-target networks: 1) an illicit drug-target network, which includes illicit drugs and their target genes; 2) an illicit-extended drug-target network, including illicit drugs, illicit drugs' targets, and illicit-related drugs; 3) a full drug-target network including illicit drugs, non-illicit drugs, and their targets from DrugBank.

Nodes that act as hubs in subnetwork or bridging nodes among subnetworks might play critical roles in drug actions [13]. Node degree, the number of edges linked to a node, is a basic but informative measure of networks' characteristics [30]. Nodes with higher degree are referred to hubs, suggesting their critical roles in the network. To identify hubs, we first calculated the degree of each node and then plotted the degree of all nodes. Based on this degree distribution, the point where the distribution begins to reach its asymptote is detected. The nodes, including drugs and genes, with a degree higher than the detected point are considered hubs.

Bridge nodes can be assessed and determined by calculating the betweenness centrality. Betweenness can measure how signals travel through the interaction network, with high betweenness reflecting multiple paths between nodes and low betweenness indicating few paths [31]. For a given node v , its betweenness is defined as the sum, for each pair of nodes $\left(s,t\right)$ in the network, of the ratio between the number of shortest paths from s  to t  passing through v  and the total number of shortest paths from s  to t . This betweenness calculation is represented mathematically for a given node v  as $B\left(v\right)=\underset{s\neq t\neq v}{\sum }\frac{\sigma_{st}\left(v\right)}{\sigma_{st}}$, where $\sigma_{st}$ represents the number of shortest paths from node s  to node t  and $\sigma_{st}\left(v\right)$ represents the number of shortest paths from node s  to node t  that also pass through node v . To identify the bridge nodes based on the betweenness, we adopted the method above used to determine hubs. We first calculated the betweenness using algorithms implemented in the Cytoscape plugin cyto-Hubba (v. 1.6) [32] and then drew the betweenness distribution to find the point that the distribution begins to reach its asymptote. The nodes, including drugs and genes, with a betweenness higher than the detected point were regarded as bridge nodes.

The networks were visualized by the software Cytoscape, an open source network analysis platform [33].

## Results

### Illicit drugs and their targets

We obtained 188 illicit drugs according to the "illicit" status annotated by DrugBank. Those illicit drugs can be grouped into 60 categories according to DrugBank category annotation. The top 10 categories of these medications are opioid analgesics (number of drugs: 24), hypnotics and sedatives (24), narcotics (21), anti-anxiety agents (19), GABA modulators (15), analgesics (13), hallucinogens (12), benzodiazepines (11), anticonvulsants (10), and central nervous system stimulants (10). These categories indicate that the illicit drugs have the potential for strong neurologic impact.

Among the 188 illicit drugs, only 86 (45.7%) have unique human protein targets, which can be mapped to 73 unique human genes. We applied the GO Slim Classification tool implemented in the WebGestalt to examine the number distribution of the 73 illicit drugs' target genes among each GO Slim term as compared to that among the 1235 non-illicit drugs' target genes. We tested GO Slim terms in two GO domains: Biological Process and Molecular Function. For each GO Slim term, we performed a Fisher's exact test and then applied a cutoff p-value of 0.05 to identify GO Slim terms enriched in a set of target genes. We identified seven GO Slim terms that are significantly enriched in the illicit target genes (Table 1). Most of them are directly related to molecular transduction, transporter activity, and ion binding. It should be noted that most of the non-illicit drugs' target genes (902, 73.0%) are involved in "metabolic process," which is much higher than the percentage of illicit drugs' target genes (33, 45.2%) in that category. Additionally, some GO Slim terms such as "nucleotide binding," "hydrolase activity," and "transferase activity" comprise approximately 15-20% of the non-illicit drugs' target genes but jointly have only one illicit drugs' target gene. These results indicate that, compared to non-illicit drugs' target genes, the illicit drugs' target genes tend to be involved in the receptor process and signaling transduction.

**Table 1:** Gene Ontology (GO) terms significantly enriched in illicit drugs' target genes compared to non-illicit drugs' target genes in the full drug-target network

GO ID	GO term^a^	# of illicit drugs' target genes (%^b^)	# of non-illicit drugs' target genes (%^c^)	p-value^d^
GO:0060089	MF: Molecular transducer activity	55 (75.3)	281 (22.8)	< 2.2 × 10^-16^
GO:0007154	BP: Cell communication	58 (79.5)	441 (35.7)	1.5 × 10^-13^
GO:0005215	MF: Transporter activity	38 (52.1)	215 (17.4)	9.9 × 10^-11^
GO:0065007	BP: Biological regulation	63 (86.3)	628 (50.9)	7.9 × 10^-10^
GO:0032501	BP: Multicellular organismal process	50 (68.5)	509 (41.2)	7.7 × 10^-6^
GO:0051179	BP: Localization	45 (61.6)	461 (37.3)	5.8 × 10^-5^
GO:0043167	MF: Ion binding	35 (47.9)	432 (35.0)	0.032

^a^Gene Ontology domains: BP (biological process) and MF (molecular function).

^b^The proportion of illicit drugs' target genes belong to a given GO Slim term among the 73 illicit drugs' target genes.

^c^The proportion of non-illicit drugs' target genes belong to a given GO Slim term among the 1235 non-illicit drugs' target genes.

^d^p-value was calculated by Fisher's exact test.

Pathway enrichment analyses were further conducted to identify over-represented canonical biological pathways among the illicit drugs' target genes compared to the other drugs' target genes. Using WebGestalt, nineteen KEGG pathways were identified as being significantly enriched with the 73 illicit drugs' target genes and 163 significant pathways enriched with the non-illicit drugs' target genes (adjusted p-value < 0.05). Then we performed the Fisher's exact test by comparing illicit and other drugs' target genes for each of the nineteen KEGG pathways (Additional file 1). Four KEGG pathways were identified as being significantly enriched in the illicit drugs' target genes. They are "neuroactive ligand-receptor" (Fisher's exact test, p-value = 2.2 × 10^-16^), "calcium signaling pathway" (p-value = 2.8 × 10^-4^), "amyotrophic lateral sclerosis" (p-value = 2.9 × 10^-3^), and "long-term potentiation" (p-value = 2.9 × 10^-3^). As expected, except for the "calcium signaling pathway," all other three pathways are directly related to neurodevelopment, which is consistent to the neuroscience theories of drug addiction. And calcium signaling has been studied with their roles in the pathogenesis of neurodevelopmental disorders.

### Illicit drug-target network

To investigate the organization and association between the illicit drugs and their targets, we first constructed an illicit drug-target network by compiling the associations between illicit drugs and their targets. For comparison purpose, we built a full drug-target network that included all drugs (illicit and non-illicit drugs), and their targets from DrugBank.

**Figure 1:**
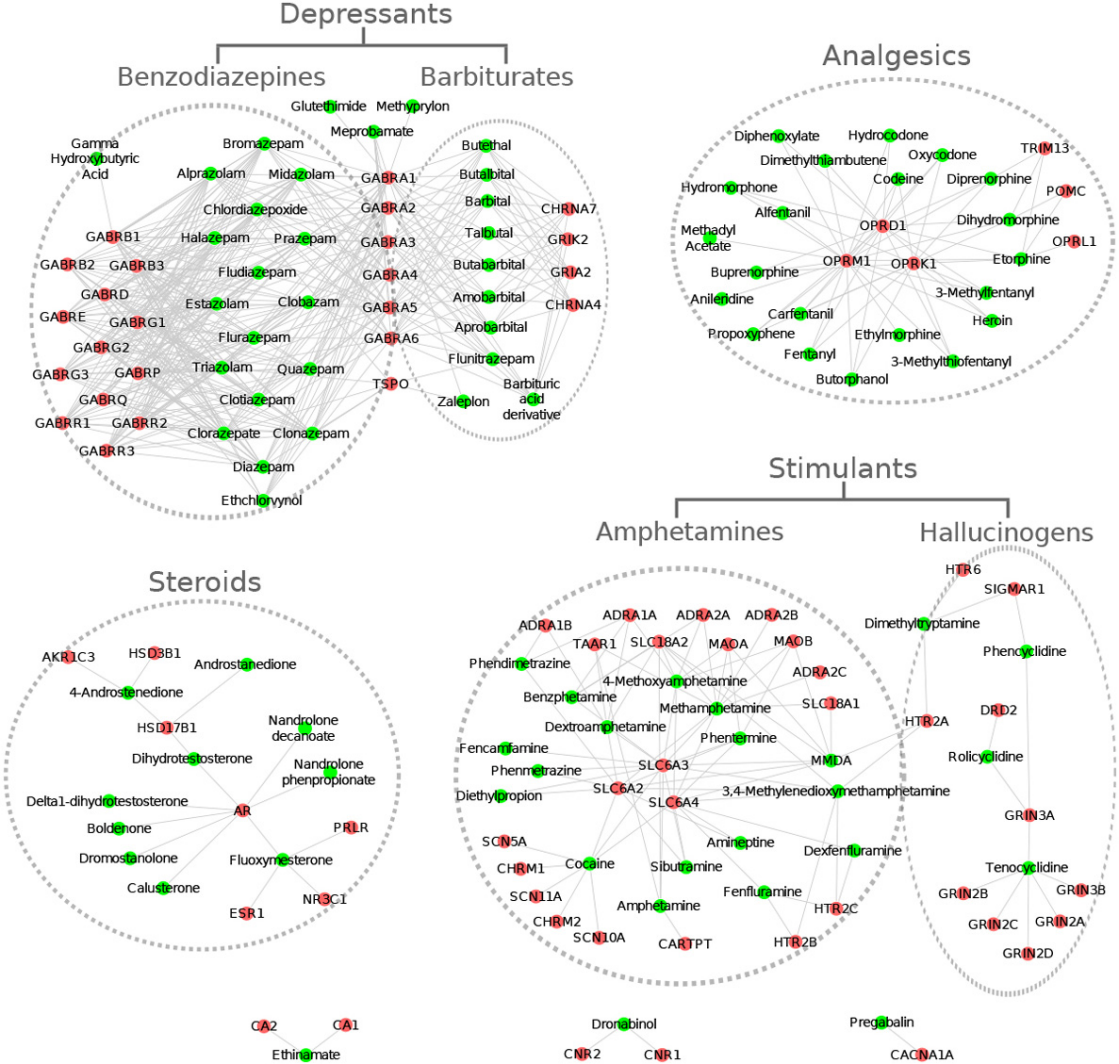
**Illicit drug-target network**. The illicit drug-target network consists of four major subnetworks corresponding to four major medication categories: depressants, analgesics, steroids, and stimulants. There are three small (orphan) subnetworks located in the bottom of the figure. Nodes in green correspond to illicit drugs and nodes in red correspond to target genes.

The illicit drug-target network has 159 nodes (86 illicit drugs and 73 target genes) and 563 edges (Additional file 2). After superimposing the DrugBank categories on the network, four topological subnetworks were identified in correspondence with four major medication categories: depressants (benzodiazepines and barbiturates), stimulants (amphetamines, hallucinogens), analgesics, and steroids (Figure 1). To further assess the classification characteristics of these drugs, we compared the ATC therapeutic second sublevels for the illicit and other drugs using Fisher's exact test. Among the 86 illicit drugs, 50 drugs have at least one ATC second sublevel classifications. And among the 773 other drugs, 594 drugs have at least one ATC second sublevel classification. We found that those drugs are over-represented in seven ATC second sublevel categories (Table 2). As expected, five of seven ATC categories belong to the nervous system and the most significant ATC category is psycholeptics (N05).

**Table 2:** ATC second sublevel categories significantly enriched in illicit drugs compared to other drugs^a^

ATC code	ATC therapeutic subgroup	# of illicit drugs (%^b^)	# of other drugs (%^c^)	p-value^d^
N05	Psycholeptics	24 (48.0)	3 (0.5)	1.2 × 10^-26^
N01	Anesthetics	5 (10.0)	0 (0)	2.3 × 10^-6^
A08	Antiobesity preparations, excluding diet products	4 (8.0)	1 (0.2)	1.5 × 10^-4^
N02	Analgesics	6 (12.0)	11 (1.9)	1.0 × 10^-3^
A14	Anabolic agents for systemic use	2 (4.0)	0 (0)	5.9 × 10^-3^
N06	Psychoanaleptics	3 (6.0)	4 (0.7)	0.012
R05	Cough and cold preparations	2 (4.0)	1 (0.2)	0.017

^a^The other drugs represent the drugs in the full drug-target network excluding illicit drugs and illicit-related drugs. Among those other drugs, 594 have ATC second sublevel classifications.

^b^The proportion of illicit drugs belong to a given ATC category among the 50 illicit drugs with ATC second sublevel classifications.

^c^The proportion of other drugs belong to a given ATC category among the 594 other drugs with ATC second sublevel classifications.

^d^p-value was calculated by Fisher's exact test.

The full drug-target network includes 2619 nodes (1286 drugs and 1333 target genes) and 5098 edges (Additional file 3). In the illicit drug-target network, 86 illicit drugs have an average of 6.6 targets (range: 1 ~ 20) while 73 target genes are targeted by 7.7 drugs on average (range: 1 ~ 29). In the full drug-target network, drugs have an average of 4.0 targets (range: 1 ~ 144) while targets are targeted by an average of 3.8 drugs (range: 1 to 76). Thus, both drug degree (number of targets) and target degree (number of drugs) in the illicit drug-target network are significantly higher than those of the full drug-target networks (Wilcoxon rank-sum test p-values for drug degree and target degree: 6.0 × 10^-4 ^and 2.4 × 10^-7^, respectively). This comparison reveals that, relative to all drug-target networks, the illicit drugs and targets tend to be strongly connected and form tight clusters (Additional file 4), which is consistent with the previous result showing that neurological drugs cluster together [34].

**Figure 2:**
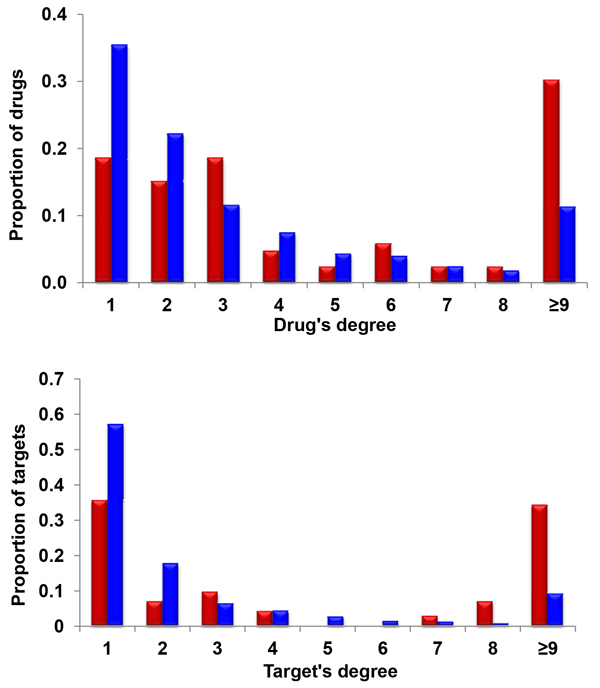
**Distribution of drugs (top panel) and drug's targets (bottom panel) by their interaction degree in illicit drug-target network (red) and the full drug-target network (blue)**. On the top panel, it shows the proportion of drug (Y-axis) measured by their degree of interactions (number of targets) (X-axis) in the network. On the bottom panel, it shows the proportion of targets (Y-axis) measured by their degree of interactions (number of drugs) (X-axis) in the network.

Using degree distribution methods, we pinpointed the nodes with a degree greater than eight as hubs for both the illicit drug-target network and full drug-target network (Additional file 5). Thus, in the illicit drug-target network, 26 drugs (30.2%) and 25 targets (34.2%) are hubs, while in the full drug-target network, 145 drugs (11.3%) and 121 targets (9.1%) are hubs (Figure 2). The proportion of hubs in the illicit drug-target network is higher than that in the full drug-target network, which is consistent with our above described results of average degree.

Of the 26 hub drugs in the illicit drug-target network, 25 belong to the cluster of depressants (alprazolam, amobarbital, aprobarbital, barbital, barbituric acid derivative, bromazepam, butabarbital, butalbital, butethal, chlordiazepoxide, clobazam, clonazepam, clorazepate, clotiazepam, diazepam, estazolam, ethchlorvynol, fludiazepam, flurazepam, halazepam, midazolam, prazepam, quazepam, talbutal, triazolam) while the other hub drug is methamphetamine. This distribution indicates that the hub drugs belong to similar categories and act via common mechanisms. Among the 26 drugs, 23 belong to psycholeptics (N05), one belong to antiepileptic (N03), and two (butabarbital and butalbital) do not have an ATC code. Among the 23 psycholeptics, thirteen are hypnotics and sedatives (N05C) while the other ten drugs are anxiolytics (N05B).

Remarkably, among the 25 hub target genes, nineteen belong to the gamma-aminobutyric acid receptors (GABRs) and act as hubs in the cluster of depressants. They are *GABRA1*, *GABRA2*, *GABRA3*, *GABRA4*, *GABRA5*, *GABRA6*, *GABRB1*, *GABRB2*, *GABRB3*, *GABRD*, *GABRE*, *GABRG1*, *GABRG2*, *GABRG3*, *GABRP*, *GABRQ*, *GABRR1*, *GABRR2*, and *GABRR3*. Three opioid receptors (*OPRD1*, *OPRK1*, and *OPRM1*) act as hubs for analgesic pharmaceuticals. Three adrenergic transporters (*SLC6A2*, *SLC6A3*, and *SLC6A4*) act as hubs in the cluster of amphetamine stimulants. These results indicate that those genes play important roles in the molecular mechanisms of drug addiction development for their corresponding drug categories.

### Illicit-extended drug-target network

To identify some medications that might have associations with illicit drugs, we explored a set of illicit-related drugs that share at least one common target with at least one illicit drug. Those drugs could have novel potential in treating or modulating drug addiction and other psychiatric ailments or might have addictive effects due to their shared set of targets. Based on their interactions with illicit drugs' targets, we added these illicit-related drugs to the illicit drug-target network to build an illicit-extended drug-target network. The illicit-extended drug-target network includes 586 nodes and 1725 edges (Additional file 6). The 586 nodes include 86 illicit drugs, 427 illicit-related drugs, and 73 illicit drugs' target genes. The average drug degree (number of target genes) is 3.4 (range: 1~20) while the average target degree (number of drugs) is 23.6 (range: 1:74). These degrees are not comparable to those of the illicit drug-target network due to the relative oversaturation of drug nodes as compared to gene nodes.

Among the 427 illicit-related drugs, 365 have at least one ATC second sublevel classification. Compared to 594 other drugs with ATC second sublevel classification, these illicit-related drugs are significantly over-represented in 12 ATC second sublevel categories (Table 3). Among them, five belong to the nervous systems (N), i.e., psycholeptics (N05), psychoanaleptics (N06), anesthetics (N01), anti-Parkinson drugs (N04), and other nervous system drugs (N07). The others belong to the corticosteroids and dermatological preparations (D07), sex hormones and modulators of the genital system (G03), corticosteroids for systemic use (H02), drugs for functional gastrointestinal disorders (A03), vasoprotectives (C05), nasal preparations (R01), and ophthalmologicals (S01) categories. Compared to the ATC second sublevel categories that are over-represented in the illicit drugs, the proportion of drugs belonging to nervous systems in the illicit-related drugs (171, 46.8%) is significantly lower than that of illicit drugs (39, 78.0%) (Fisher's exact test, p-value = 3.4 × 10^-5^). These results revealed, though most of the illicit-related drugs still belong to the nervous systems, they also involve more drugs from other categories.

**Table 3:** ATC second sublevel categories significantly enriched in illicit-related drugs compared to other drugs^a^

ATC code	ATC therapeutic subgroup	# of illicit-related drugs (%^b^)	# of other drugs (%^c^)	p-value
N05	Psycholeptics	54 (14.8)	3 (0.5)	1.4 × 10^-20^
N06	Psychoanaleptics	38 (10.4)	4 (0.7)	6.7 × 10^-13^
N01	Anesthetics	25 (6.9)	0 (0)	1.9 × 10^-11^
D07	Corticosteroids, dermatological preparations	23 (6.3)	0 (0)	1.4 × 10^-10^
N04	Anti-Parkinson drugs	17 (4.7)	2 (0.3)	4.2 × 10^-6^
G03	Sex hormones and modulators of the genital system	21 (5.8)	5 (0.8)	1.1 × 10^-5^
H02	Corticosteroids for systemic use	12 (3.3)	1 (0.2)	7.0 × 10^-5^
N07	Other nervous system drugs	14 (3.8)	5 (0.8)	2.9 × 10^-3^
A03	Drugs for functional gastrointestinal disorders	14 (3.8)	5 (0.8)	2.9 × 10^-3^
C05	Vasoprotectives	12 (3.3)	4 (0.7)	3.2 × 10^-3^
R01	Nasal preparations	16 (4.4)	7 (1.2)	3.6 × 10^-3^
S01	Ophthalmologicals	43 (11.8)	44 (7.4)	0.027

^a^The other drugs represent the drugs in the full drug-target network excluding illicit drugs and illicit-related drugs. Among those other drugs, 594 have ATC second sublevel classifications.

^b^The proportion of illicit drugs belong to a given ATC category among the 365 illicit-related drugs with ATC second sublevel classifications.

^c^The proportion of other drugs belong to a given ATC category among the 594 other drugs.

^d^p-value was calculated by Fisher's exact test.

**Figure 3:**
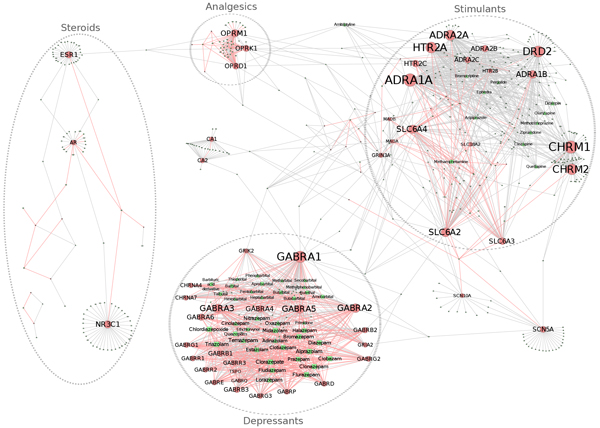
**Illicit-extended drug-target network, highlighted with hubs**. Nodes with readable labels are hubs, which were defined by their degrees being greater than eight. Rectangular nodes in green correspond to illicit drugs, triangular nodes in green correspond to illicit-related drugs, and circle nodes in red correspond to target genes. Red edges represent the interactions between illicit drugs and their target genes and grey edges represent the interactions between illicit-related drugs and their target genes that are also targeted by illicit drugs. Font size corresponds to the degree of interactions.

The nodes in the illicit-extended drug-target network with degrees greater than eight are defined as hubs, according to the degree distribution methodology. Of the 427 illicit-related drugs in the network, 26 are hubs (Figure 3). Among the 26 drugs, 22 have ATC classifications. While all 22 drugs belong to the main nervous system (N) category, they have a more diverse range of therapeutic subgroups. For example, fifteen are psycholeptics (N05), three are antiepileptics (N03), two are anti-Parkinson (N04), and two others are psychoanaleptics (N06). Among the 73 target genes, 52 have their degrees greater than eight. This is primarily due to the relative oversaturation of drugs in the illicit-extended drug-target network. In addition to the 25 genes that already are hubs in the illicit drug-target network, an additional 27 genes act as hubs because of the inclusion of targeting non-illicit drugs. For example, gene *CHRM1*is targeted by one illicit drug and 71 non-illicit drugs. Other examples include *DRD2 *(number of illicit drugs: 1; number of non-illicit drugs: 62), *CHRM2 *(number of illicit drugs: 1; number of non-illicit drugs: 59), *NR3C1 *(number of illicit drugs: 1; number of non-illicit drugs: 37), and *ADRA2B *(number of illicit drugs: 1; number of non-illicit drugs: 31).

**Figure 4:**
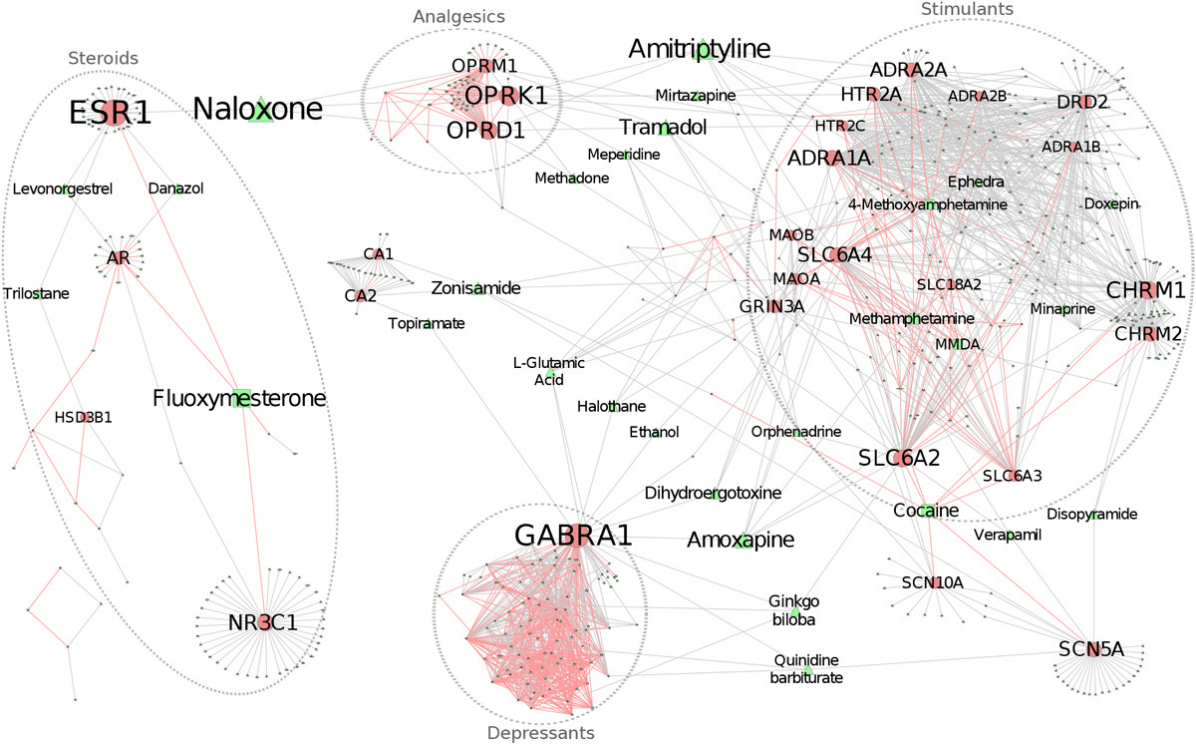
**Illicit-extended drug-target network, highlighted with bridge nodes**. Nodes with readable labels are bridge nodes, whose betweenness value is greater than 2000. Font size corresponds to the betweenness value. Colors and shapes of nodes as well as colors of edges are defined as in Figure 3.

In addition to identifying hubs, we also sought out bridge nodes by computing a betweenness value for each node in the illicit-extended drug-target network. To categorize bridge nodes, we used a method similar to the degree distribution and assessed betweenness values by their rank (Additional file 7). We determined that a node with the betweenness value greater than 2000 as bridge nodes. There are 51 bridge nodes (Figure 4), in which 25 belong to drugs and 26 belong to target genes. Among the 25 drug bridge nodes, only two exist in the 26 drug hubs. Thus, we identified 49 drugs that might have high potentials for addiction effects or efficient treatment of addiction. Among the 73 target genes, 24 act as bridge nodes. Among them, gene *ESR1 *links the cluster of analgesics to the cluster steroids and gene *GABRA1 *links the cluster of depressants to the cluster stimulants.

## Discussion

In this study, we explored the relationships among illicit drugs, illicit-related drugs, and their targets in the context of networks in order to further understand the molecular mechanisms of addiction. We presented a systematic method for studying illicit drugs and their targets, even for those non-illicit drugs that share common targets with illicit drugs. Furthermore, we have shown that network topological analyses used to identify important nodes could serve as a basic tool for knowledge discovery.

One important output of this study is the illicit drug-target network. Though the network is not very complex, its clustering can capture a drug classification pattern, which provides evidence that the network pharmacology approach is effective and executable to investigate medications and their targets. In the network, the 86 illicit drugs with 73 target genes tend to cluster together, forming four subnetworks corresponding to four medication groups: depressants, stimulants, analgesics, and steroids. Further external analysis of ATC codes confirmed that the illicit drugs have neurological functions or act via mechanisms of stimulants, opioids, and steroids.

Another important application of network analysis in this study is to recruit the non-illicit drugs into the illicit drug-target network, which demonstrates the identification of potential drug repositioning using network analysis [35]. Due to the disjointed nature of the subnetworks and the relative skew of hub nodes in the depressants subnetwork, an extended network is necessary to identify further modulators of addiction. The illicit drug network was extended by including all illicit and non-illicit drugs that targeted any illicit drugs' target. As a result, the network was oversaturated with pharmaceuticals and this step allowed for new connections between subnetworks and an improvement in the possibility of identifying important drugs that modulate similar sets of genes upon which illicit drugs act. Most of the illicit-related drugs in this network have some neurological function or act through a steroid or neurotransmitter mechanism. The power of this extended network is to identify the hub and bridge nodes that might play important roles for addiction development or treatments. For example, naloxone, the strongest drug bridge node, is an opioid inverse agonist which is used to treat opioid overdoses and counteract emergent depressant of vital functions [36,37]. The opioid receptor OPRK1, the third strongest bridge node, has been investigated as a potential target in the treatment of drug addiction, possibly via dynorphin [38-40]. The seventh strongest bridge node, glucocorticoid receptor NR3C1, has been studied as a possible neuroendocrine mediator of stress and addiction response [41,42]. Aripiprazole, a hub, is frequently used to treat methamphetamine abuse [43] and alcohol dependence abuse [44] and bromocriptine, another hub, is used in the treatment of cocaine addiction [45]. It is also interesting to observe that some nodes that connect various subnetworks. For example, l-glutamic acid, ethanol, halothane, dihydrogenotoxine, amoxapine, ginko biloba, quinidine barbiturate, SLC6A2, and GRIN3A connect the clusters of depressants and stimulants. Amitriptyline, mirtazapine, tramadol, meperidine, methadone, HTR2C, and HTR2A, ADRA2A connect the analgesics and stimulants. The depressants and analgesics are interestingly connected via nodes in the analgesics, stimulants, and depressants. Finally, naloxone and ESR1 connect the analgesics to the steroids. These connection patterns could help focus future research based on the important nodes identified through this type of systematic analysis.

Recently, Li and Burmeister [5] compiled 62 candidate genes associated with at least one drug addiction based on verified genetic evidence. Among the candidate genes, 47 have been targeted by at least one of the 1286 drugs and 17 have been targeted by at least one of the illicit drugs. Thus, comparing the 1333 genes targeted by these 1286 drugs, Li and Burmeister's gene set is significantly enriched in the illicit drug-target network (hypergeometric test, p-value = 3.9 × 10^-11^). This finding illustrates another link between genetic factors and drugs' targets in addiction development. Additionally, among the 17 genes, five (*GABRA2*, *OPRD1*, *OPRK1*, *OPRM1*, and *SLC6A2*) are hubs, which indicates that proteins vital to the mediation of neurological function such as GABA receptors, opioid receptors, and adrenergic transporters act as the main hub genes of the illicit drug network.

In this study, we extracted the interaction data between drugs and their targets mainly from DrugBank. Though the study provides a systematic review of relationships among illicit drugs, their targets, and illicit-related drugs, it still needs to be improved since the current data utilized in this study is neither complete nor error- or bias-free. For example, among 188 illicit drugs, only 86 drugs have protein-coding targets. This indicates that further investigation is needed to illustrate the molecular mechanisms of drug addiction. Future research in this area should include more drug-target information from multiple target-centered databases such as Matador and SuperTarget [46] and the Therapeutic Target Database (TTD) [47,48]. Additionally, a large volume of genome-wide molecular neuropharmacology data, such as microarray gene expression, genome-wide association studies, and next-generation sequencing data, is available, and much more data will become available in the near future due to the rapid advances in high throughput technologies and the strong efforts supported by National Institutes of Health, numerous other foundations, and pharmaceutical industry. Therefore, it is possible and necessary to develop novel methods for multi-dimensional data integration at the network and pathway levels, and beyond, so that these studies will improve the accuracy and coverage of identification of drug's target nodes and drug nodes. To this end, novel subnetwork search and module finding algorithm development and their applications to this field is required. There are many network algorithms available [49,50], which can be adopted or enhanced for network pharmacology studies. In summary, the network pharmacology approach, combining with multi-dimensional data, would allow researchers to paint a more comprehensive view of addiction genetics and identify a greater portion of interconnections between drugs and genes.

## Conclusion

This study is important because of the burden that abused drugs place on individuals, their families, the American healthcare system, and global health and societal influence. The emerging area of network pharmacology may be able to identify new treatments for managing illicit drug abuse. However, more work is necessary to identify common patterns to extract interactions and influence treatment. Potential findings could be used to inform treatment strategies based on patient genetics, allow for secondary uses of drugs that have the same targets, and improve treatments for drug addiction, which in turn may aid psychiatric care and reduce the burden on emergency rooms.

## Competing interests

The authors declare that they have no competing interests.

## Authors' contributions

RVA and JS collected the data and conducted data analysis. RVA, JS and ZZ conceived and designed the study. RVA, JS and ZZ wrote the manuscript. All authors read and approved the final manuscript.

## Supplementary Material

Additional file 1 **KEGG pathways significantly enriched in illicit drugs' target genes compared to non-illicit drugs' target genes**. Click here for file

Additional file 2 **List of pairs in illicit drug-target network**. Click here for file

Additional file 3 **List of pairs in full drug-target network**. Click here for file

Additional file 4 **Mapping the illicit drug-target network into the full drug-target network**. Blue nodes represent target genes while grey nodes represent drugs. The red edges are the interactions between illicit drugs and their target genes while the grey edges are the interactions between non-illicit drugs and their target genes. Click here for file

Additional file 5 **Degree distribution of illicit drug-target network (left) and full drug-target network (right)**. The red line indicates the degree cutoff value (9) for definition of hubs. Click here for file

Additional file 6 **List of pairs in the illicit-extended drug-target network**. Click here for file

Additional file 7 **Betweenness distribution of the illicit-extended drug-target network**. The red line indicates the betweenness cutoff value (2000) for definition of bridge nodes. Click here for file

## Declarations

The publication costs for this article were funded by the corresponding author.

This article has been published as part of *BMC Genomics *Volume 14 Supplement S4, 2013: Selected articles from the IEEE International Conference on Bioinformatics and Biomedicine 2012: Genomics. The full contents of the supplement are available online at http://www.biomedcentral.com/bmcgenomics/supplements/14/S4.
