# Supplementary figures and images for: Exploring drug-target interaction networks of illicit drugs
Source: BMC Genomics. 2013 Oct 1;14(Suppl 4):S1. doi: 10.1186/1471-2164-14-S4-S1 (PMC3849475; doi:10.1186/1471-2164-14-S4-S1)

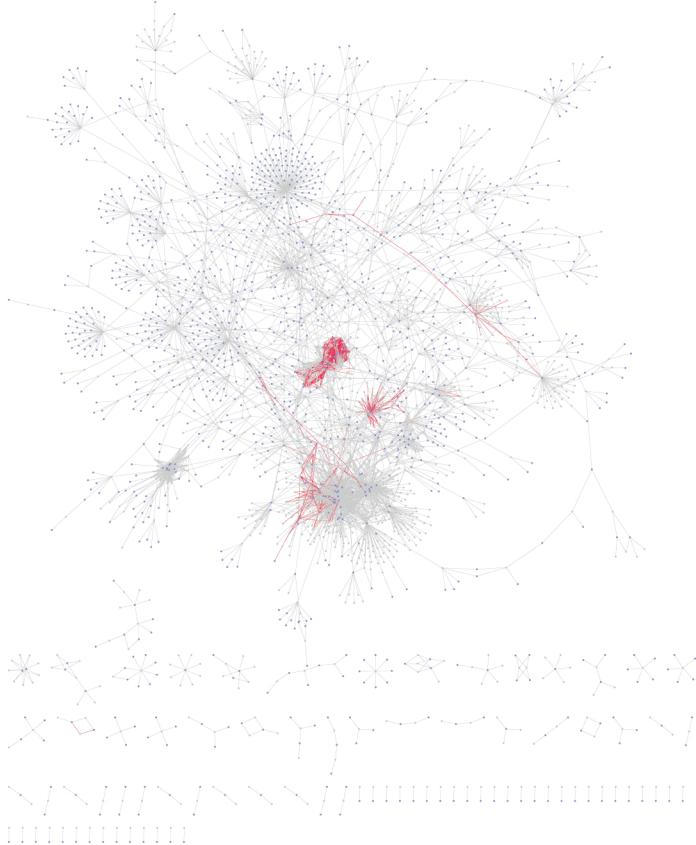

Supplement: Additional file 4 — Mapping the illicit drug-target network into the full drug-target network. Blue nodes represent target genes while grey nodes represent drugs. The red edges are the interactions between illicit drugs and their target genes while the grey edges are the interactions between non-illicit drugs and their target genes. [file 1471-2164-14-S4-S1-S4.pdf]

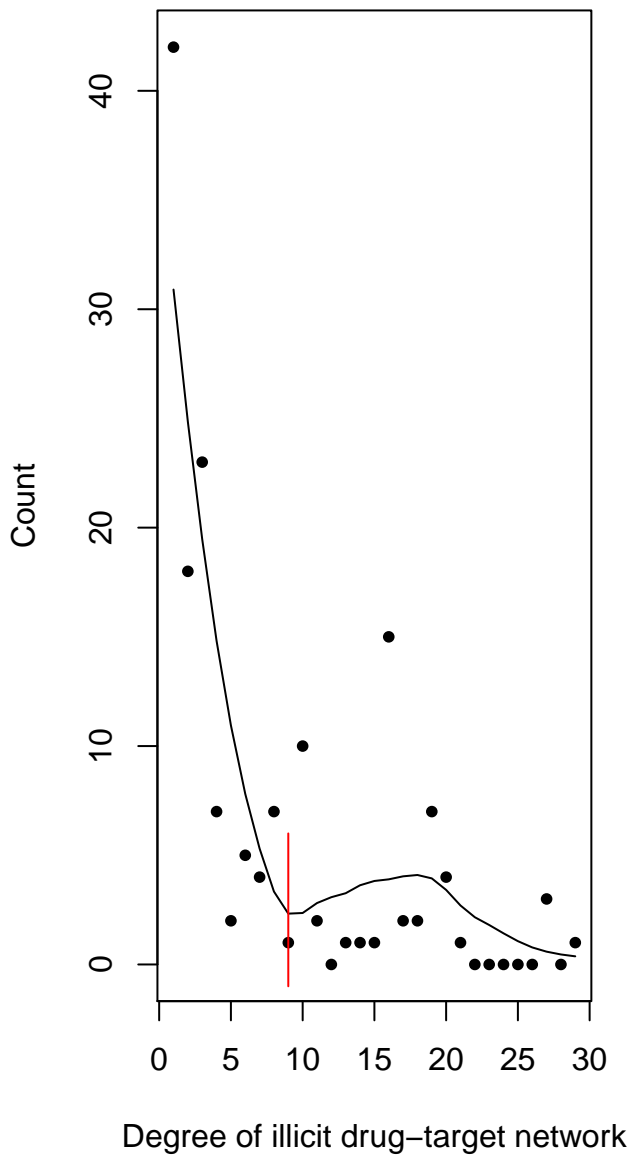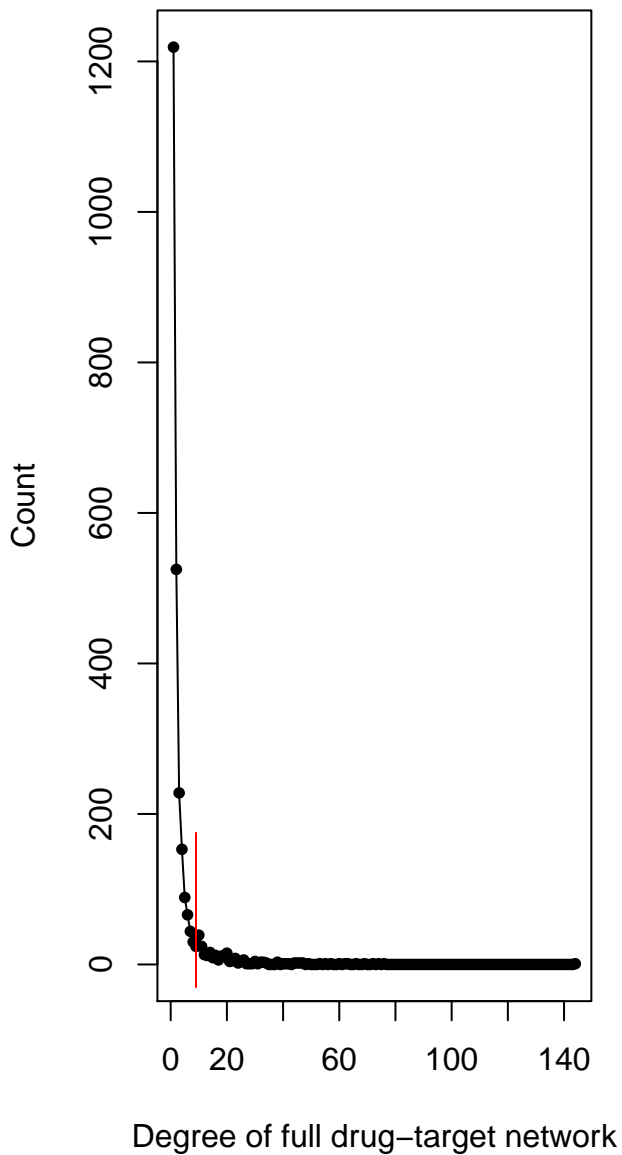

Supplement: Additional file 5 — Degree distribution of illicit drug-target network (left) and full drug-target network (right). The red line indicates the degree cutoff value (9) for definition of hubs. [file 1471-2164-14-S4-S1-S5.pdf]

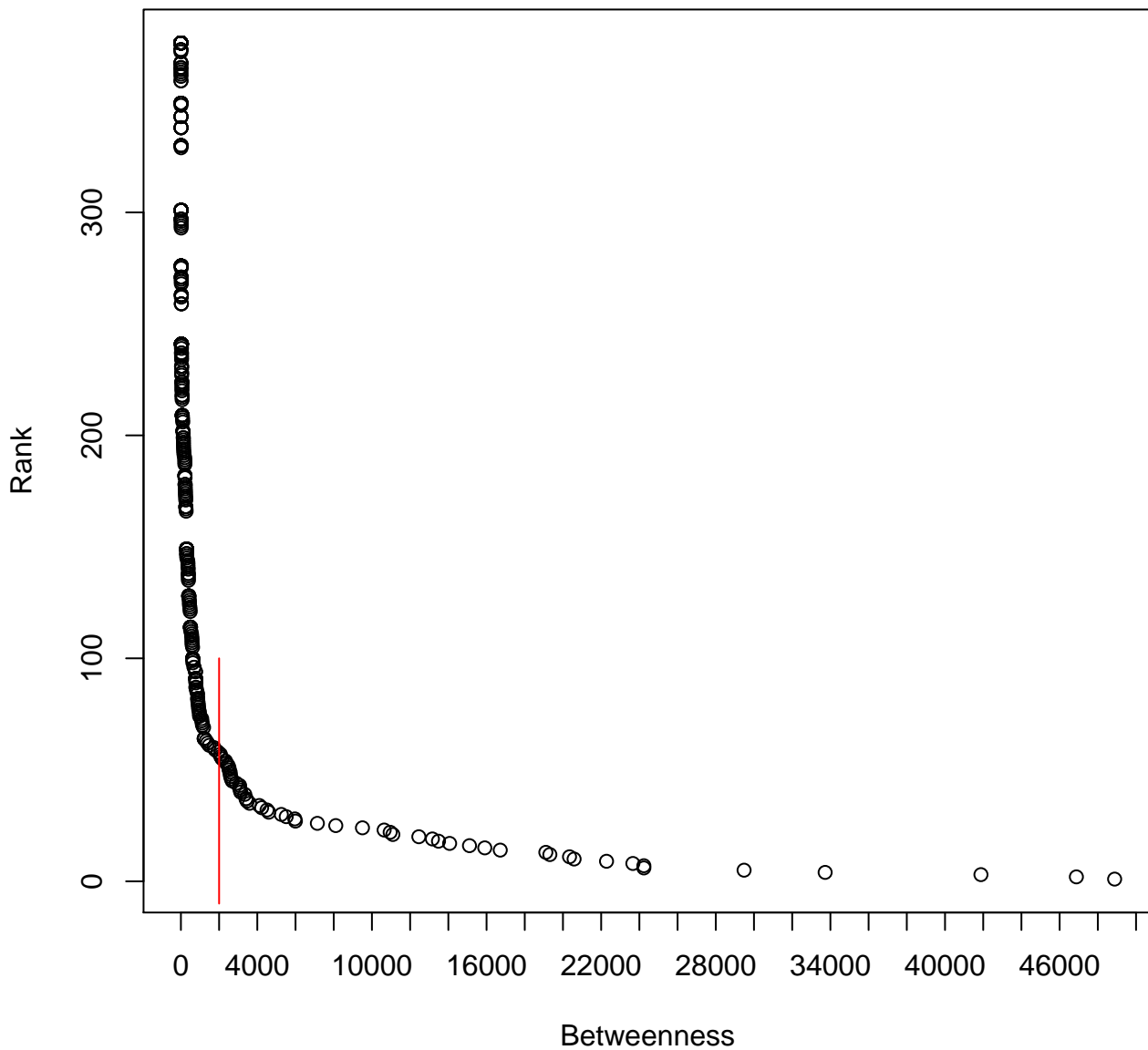

Supplement: Additional file 7 — Betweenness distribution of the illicit-extended drug-target network. The red line indicates the betweenness cutoff value (2000) for definition of bridge nodes. [file 1471-2164-14-S4-S1-S7.pdf]
